# A Novel Bufalin Derivative Exhibited Stronger Apoptosis-Inducing Effect than Bufalin in A549 Lung Cancer Cells and Lower Acute Toxicity in Mice

**DOI:** 10.1371/journal.pone.0159789

**Published:** 2016-07-26

**Authors:** Miao Liu, Li-Xing Feng, Peng Sun, Wang Liu, Wan-Ying Wu, Bao-Hong Jiang, Min Yang, Li-Hong Hu, De-An Guo, Xuan Liu

**Affiliations:** Shanghai Institute of Materia Medica, Chinese Academy of Sciences, Shanghai 201203, P.R. China; Universidade Federal do Rio de Janeiro, BRAZIL

## Abstract

BF211 is a synthetic molecule derived from bufalin (BF). The apoptosis-inducing effect of BF211 was stronger than that of BF while the acute toxicity of BF211 was much lower than that of BF. BF211 exhibited promising concentration-dependent anti-cancer effects in nude mice inoculated with A549 cells *in vivo*. The growth of A549 tumor xenografts was almost totally blocked by treatment with BF211 at 6 mg/kg. Notably, BF and BF211 exhibited differences in their binding affinity and kinetics to recombinant proteins of the α subunits of Na+/K+-ATPase. Furthermore, there was a difference in the effects of BF or BF211 on inhibiting the activity of porcine cortex Na+/K+-ATPase and in their time-dependent effects on intracellular Ca2+ levels in A549 cells. The time-dependent effects of BF or BF211 on the activation of Src, which was mediated by the Na+/K+-ATPase signalosome, in A549 cells were also different. Both BF and BF211 could induce apoptosis-related cascades, such as activation of caspase-3 and the cleavage of PARP (poly ADP-ribose polymerase) in A549 cells, in a concentration-dependent manner; however, the effects of BF211 on apoptosis-related cascades was stronger than that of BF. The results of the present study supported the importance of binding to the Na+/K+-ATPase α subunits in the mechanism of cardiac steroids and also suggested the possibility of developing new cardiac steroids with a stronger anti-cancer activity and lower toxicity as new anti-cancer agents.

## Introduction

**Fig 1:**
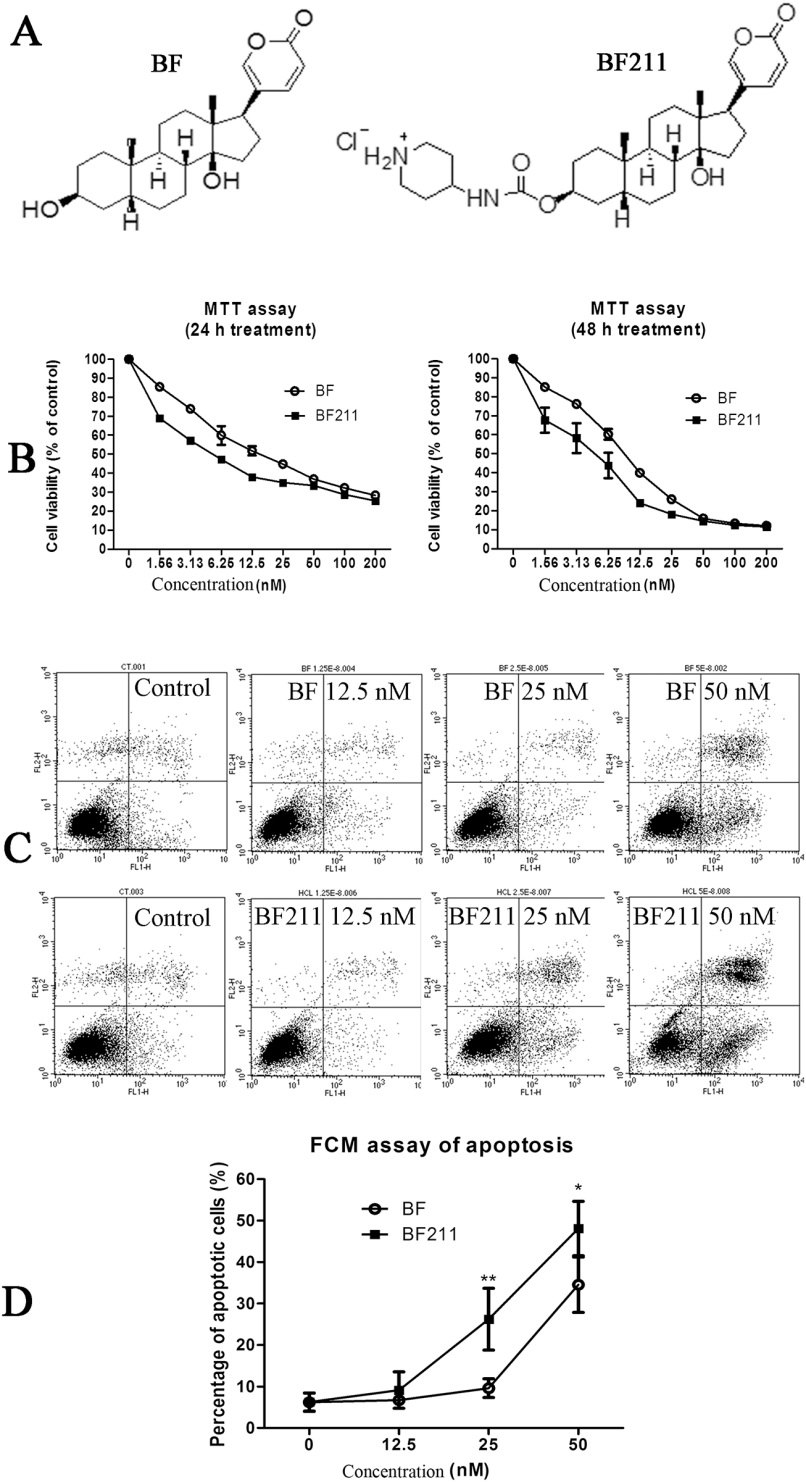
Inhibiting effects of BF and BF211 on the proliferation of A549 cells. (A) Chemical structures of BF and BF211. (B) The cell viability (MTT assay result) of A549 cells treated with various concentrations of BF or BF211 for 24 or 48 h. The data comprise the statistical results of three independent experiments. (B) A representative of the flow cytometry analysis results of apoptosis induced by 24 h treatment of BF or BF211 at different concentrations. (D) The statistical analysis results of the percentage of apoptotic cells after treatment with BF or BF211 at different concentrations for 24 h. The data comprise the statistical results (n = 3, mean ± SEM) of three independent experiments. *p<0.05, **p<0.01 vs. BF-treated group.

Bufalin (BF) is a bufadienolide isolated from a traditional Chinese medicine ChanSu, the dried secretion from the skin and parotid venom glands of *Bufo bufo gargarizans* Cantor or *Bufo melanostictus* Schneider [1]. ChanSu had been used to clinically treat patients with various cancers in China for a long time [2,3]. Notably, in the past two decades, the study of the anti-cancer effects of cardiac steroids, including both bufadienolides and cardenolides, has been a hot topic in the anti-cancer drug research area [4,5,6]. The research of our group has focused on studying the structure-activity relationship of bufadienolides and finding BF derivatives with better anti-cancer activity and lower toxicity [7]. Among our BF derivative collections, a compound named BF211, whose structure is shown in Fig 1, has exhibited promising results. This compound has been granted an invention patient in China (Authorized Announcement No. CN 102532235B). Similar to BF, BF211 showed potent anticancer activities to a broad spectrum of tumor cell lines with nanomolar level IC50 values [8]. In the present study, non-small-cell lung cancer A549 cells were used to further study the anti-cancer effects of BF and BF211, because ChanSu has a good efficiency in the clinical treatment of lung cancer [2,9]. Both the *in vitro* and *in vivo* anti-cancer effects of BF and BF211 on A549 cells were studied. The acute toxicity of BF211 was determined and compared with that of BF. The possible mechanisms that may contribute to the difference between the two compounds were then studied.

## Materials and Methods

### Chemicals

Bufalin (BF) was isolated from ChanSu as described previously [7] and BF211 was synthesized from BF. The chemical structures of BF and BF211 are shown in Fig 1A. Stock solutions of BF and BF211 were prepared in DMSO at a concentration of 0.1 M and then stored at -20°C.

### Cell culture

The human lung cancer A549, NCI-H460, NCI-H522 and NCI-H1299 cell lines were purchased from the Cell Resource Center of Shanghai Institutes for Biological Sciences, Chinese Academy of Sciences (Shanghai, P.R. China). The cells were cultured in RPMI 1640 medium supplemented with 10% fetal bovine serum, 100 units/mL of penicillin, and 100 μg/mL of streptomycin and maintained in a humidified chamber at 37°C containing 5% CO_2_. Fetal bovine serum, RPMI 1640, penicillin and streptomycin were all purchased from Hyclone.

### MTT assay

Cell proliferation was assessed using a colorimetric MTT assay as described previously [10]. Briefly, cells were seeded in 96-well plates at a density of 2×10^4^ (A549), 5×10^4^ (NCI-H460 and NCI-H1299) or 6×10^4^ (NCI-H522) cells/mL and then incubated overnight before treatment with BF or BF211 at different concentrations or 0.1% DMSO (solvent control) for 24 or 48 h. After treatment, the viability of cells was evaluated by checking the absorbance at 570 nm.

### FCM analysis of apoptosis

Flow cytometric analysis of the level of apoptosis was conducted using Alexa Fluor 488 Annexin V & PI /Dead Cell Apoptosis Kit (Invitrogen) according to the manufacturer’s instructions [10]. Briefly, after the 24 h treatment with BF or BF211 at different concentrations, the cells were collected, washed with PBS and resuspended in binding buffer. Then, the cells were incubated with Annexin V-FITC and propidium iodide for 15 min in the dark at room temperature, and flow cytometric analysis was conducted. Data analysis was performed with the CellQuest software.

### Acute toxicity study of BF211 and BF

A single dose toxicity study of BF211 or BF (intraperitoneally, i.p.) was conducted using adult male and female ICR mice according to Organization for Economic Co-operation and Development (OECD) Guidelines no. 423 [11]. To calculate the LD50 values of a compound, its acute toxicity at 7 concentrations needs to be checked and at least 20 mice, including 10 male and 10 female mice, have to be used for each concentration. Therefore, 140 mice were used in determining the LD50 values of BF211. Besides, 40 mice, including 20 male and 20 female mice, were used to check the acute toxicity of BF at 2 concentrations. Therefore, totally 180 mice were used in the study of acute toxicity. Briefly, 180 mice (90 males and 90 females) weighing 18–22 g were purchased from Shanghai Experimental Animal Center. Mice were divided into 18 groups of 10 mice each (10 females or 10 males) and were matched, as closely as possible, for weight and size per group. In the 18 groups, 14 groups were randomly assigned to receive a single administration of 9.4, 11.1, 13.1, 15.4, 18.1, 21.3 and 25 mg/kg of BF211 for male mice and a single administration of 9.8, 11.5, 13.6, 16.0, 18.8, 22.1 and 26 mg/kg of BF211 for female mice. Four groups of mice were randomly assigned to receive a single administration of 2.8 or 3.5 mg/kg of BF in both male mice and female mice. According to OECD Guidelines no. 423 [11], the animals were observed individually after dosing at least once during the first 30 min; periodically during the first 24 h, with special attention given during the first 3 h; and daily thereafter, for a total of 14 days, except when they were humanely killed for animal welfare reasons or were found dead. During the 3 h observation period and other monitoring time periods, animals found in a moribund condition (head and body lying flat on cage bedding with little or no response to stimuli) and animals showing severe pain or severe distress (agonal breathing, intermittent gasping or both) were humanely killed. All procedures involving animals in the present study, including the daily monitoring protocol and the criteria of humane endpoints, were reviewed and approved by the Institutional Animal Care and Use Committee of Shanghai Institute of Materia Medica, Chinese Academy of Sciences. At the end of the experiment, all surviving animals were euthanized by CO_2_ inhalation. In the experiment, 90 mice were humanely killed during the observation periods, 5 mice were found dead and 85 mice were euthanized at the end of the experiment. The cause of death in the mice found dead might also be the cardiac tonic effects of BF211, and the death may have occurred later because of the delayed absorption of BF211 (i.p.) in these mice. The LD50 value was calculated according to the Bliss method.

### Human A549 tumor xenograft experiments

Male nude immunodeficient Balb/c-nu-nu mice, aged 6–7 weeks were purchased from the Shanghai Experimental Animal Center and housed in accordance with protocols approved by the Shanghai Institute of Materia Medica, Chinese Academy of Sciences. Generally, on day 0, A549 cells (8×10^6^ cells) suspended in 0.1 mL of PBS were mixed with 0.1 mL Matrigel basement membrane matrix (BD Biosciences) and then inoculated s.c. in the right flank of each mouse. Tumors were allowed to reach 100–300 mm^3^ in size before treatments were adminstered. In the animal experiment, the tumor size was measured every 3–4 days using calipers and the tumor volume was calculated using the standard formula, width^2^×length/2. Body weight was measured daily.

Two experiments using nude mice with A549 tumor xenografts were conducted. In the first experiment, we tried to compare the anti-cancer effects of BF and BF211 at the same concentrations. Briefly, 12 days after inoculation, the mice were randomly divided into 6 groups (6 mice in each group) and began treatment with the vehicle control (natural saline, i.p., qd); a positive control (rapamycin, 10 mg/kg, i.v., once a week); BF at 0.8 mg/kg, i.p., qd; BF at 0.4 mg/kg for 7 days, which was then increased to 1.6 mg/kg for 9 days, i.p., qd; BF211 at 0.8 mg/kg, i.p., qd; BF211 at 0.4 mg/kg for 7 days, which was then increased to 1.6 mg/kg for 9 days, i.p., qd. In the second experiment, the anti-cancer effects of BF211 were determined at higher concentrations. Briefly, 15 days after inoculation, mice were randomly divided into 5 groups (10 mice in each group) and started treatment with the vehicle control (i.p., qd); a positive control (rapamycin, 10 mg/kg, i.v., once a week); BF211 at 2 mg/kg, i.p., qd; BF211 at 4 mg/kg, i.p., qd; BF211 at 6 mg/kg, i.p., qd. No mice died during this experimental period, and all animals were euthanized by CO_2_ inhalation at the end of the experiment.

### RT-PCR analysis of mRNA levels of Na+/K+-ATPase α subunits in A549 cells

The total RNA of A549 cells was extracted using TRIzol reagent (Takara) and then reverse-transcribed using the PrimeScript^™^ RT Reagent Kit (Takara). RT-PCR amplification of the cDNA was performed using primers for the Na+/K+-ATPase α subunits and the Stratagene Mx3000P Multiplex Quantitative PCR System with SYBR Premix Ex Taq^™^ (Takara). The primers used for PCR amplification of human ATP1A1 (encoding the α1 subunit), ATP1A2 (encoding the α2 subunit) and ATP1A3 (encoding the α3 subunit) were listed in S1 Table.

### The expression of the recombinant extracellular portion of the α subunits of human Na+/K+-ATPase

The recombinant extracellular portion of human Na+/K+-ATPase α1 subunit (M1-M2, M3-M4, M5-M6, M7-M8 and M9-M10) was expressed as a His-fusion protein using *Escherichia coli*, and purified by affinity chromatography. Briefly, the extracellular portion of the α1 subunit gene incorporating *BamH* I and *Hind* III sites was synthesized by Life Tech. Ltd. (Shanghai, China). The *BamH* I-*Hind* III fragment of the gene was subcloned into a pET-32b expression vector (Novagen) and transformed into *E*. *coli* BL21 (*DE3*) cells (Novagen). The BL21 transformants were cultivated in LB medium with ampicillin to an A600 nm of 0.8 at 37°C. Expression was induced by adding isopropyl β-D-thiogalactopyranoside (IPTG) to a final concentration of 10 mM, and incubated overnight at 20°C. The fusion protein containing a His-tag was isolated from the bacterial lysates by Ni^2+^-chelation affinity chromatography using a Bio-Scale^™^ Mini Profinity^™^ IMAC Cartridge (Bio-Rad). Finally, the recombinant protein was desalted using a Bio-Scale^™^ Mini Bio-Gel P-6 Desalting Cartridge (Bio-Rad). The recombinant extracellular portions of the α2 and α3 subunits of human Na+/K+-ATPase were also expressed and purified using similar methods.

### Surface plasmon resonance (SPR) biosensor analysis

The binding affinity of BF or BF211 to the recombinant proteins α1, α2, α3 subunits of Na+/K+-ATPase was assayed *in vitro* by the Drug Discovery and Design Center, Shanghai Ins titute of Materia Medica, Chinese Academy of Sciences using a SPR-based Biacore 3000 instrument (Biacore AB, Rapsgatan 7, S-754 50 Uppsala, Sweden) as reported previously [12]. Briefly, for the SPR assay, the α1, α2 or α3 subunit protein was immobilized on a CM5 sensor chip as a ligand with N-ethyl-N’-(3-dimethylaminopropyl) carbodiimide (EDC) and N-hydroxysuccinimide (NHS) according to standard primary amine-coupling procedures. The amount of immobilized protein on the chip was 11403.2 RU, 12038.9 RU and 11773.4 RU for α1, α2 and α3 subunits, respectively. HBS-EP (10 mM HEPES, 150 mM NaCl, 3 mM EDTA, and 0.005% (v/v) surfactant P20, pH 7.4) was used as the running buffer. The equilibration of the baseline was performed by a continuous flow of HBS-EP through the chip surface for 1 to 2 h. The Biacore data were collected at 25°C with HBS-EP as the running buffer at a constant flow of 30 μL/min. Samples with concentrations of BF or BF211 at 0, 2.4, 3.43, 4.90, and 7 μM were injected into the channels at a flow rate of 30 μL/min, followed by washing with the running buffer. The binding responses were recorded continuously in response units (RU) at a frequency of 1 Hz in sensorgrams and presented as a function of time. The association (*k*_a_) and dissociation (*k*_d_) rate constants and the equilibrium dissociation constant (*K*_D_ = *k*_d_ /*k*_a_) were determined by the analysis of the sensorgram-curves obtained at different concentrations of BF or BF211 using the BIA evaluation software version 3.1 (Biacore) and the 1:1 Langmuir binding fitting model.

### Effects of BF and BF211 on activities of porcine cortex Na+/K+-ATPase

The activity of Na+/K+-ATPase was determined by measuring the amount of inorganic phosphate (Pi) liberated from ATP, as described previously [13]. Briefly, a commercial Na+/K+-ATPase from porcine cerebral cortex (Sigma-Aldrich, USA) was incorporated into a reaction mixture containing 1 mM ATP, 5 mM MgCl_2_, 80 mM NaCl, 20 mM KCl, and 40 mM Tris-HCl (pH 7.8). The enzymatic reaction was terminated by adding trichloroacetic acid after the incubation period (1 h). After centrifugation, the supernatant was diluted with deionized water and then the color development reagent provided by the QuantiChrom^™^ Phosphate Assay Kit (BioAssay Systems, USA) was added. After 30 min of incubation at room temperature, the color intensity was measured at 620 nm on GENIOS reader (TEACAN, Switzerland). The Na+/K+-ATPase energy transducing activity was expressed as μmol Pi liberated from ATP by 1 mg of Na+/K+-ATPase in 1 h.

### The time-dependent effects of BF and BF211 on the free intracellular Ca2+ level of A549 cells

The level of intracellular free Ca2+ in A549 cells was determined using the fluorescent dye Fluo-3 AM (S1056, Beyotime Biotechnology, Shanghai, China), which can cross the cell membrane and be cleaved into Fluo-3 by intracellular esterase. Then, Fluo-3 can specifically combine with the free intracellular Ca2+ and exhibit a strong fluorescence at an excitation wavelength of 488 nm. After being exposed to 50 nM BF or BF211 for 0.5, 1, 10, 30, 60 and 180 min, the cells were harvested, washed twice with PBS, and then incubated with Fluo-3 AM (5 μM) in the dark at 37°C for 1 h. After washing twice with PBS to remove the remaining extracellular probe, the cells were submitted to flow cytometric analysis with an excitation wavelength of 488nm. The quantification was conducted using the FlowJo software program based on the mean values of the peak fluorescent intensity.

### Western blot assay

Western blot assays were conducted as described previously [10]. Briefly, the cellular protein samples were denatured by mixing with equal volume of 2 × sample loading buffer and then boiling at 100°C for 5 min. An aliquot (50 μg as protein) of the supernatant was loaded onto a 12% SDS gel, separated electrophoretically, and transferred to a Nitrocellulose membrane (Bio-Rad). After the membrane was incubated with 10 mM TBS with 20% Tween 20 and 5% dehydrated skim milk to block nonspecific protein binding, the membrane was incubated with primary antibodies overnight at 4°C. The primary antibodies used for detection of α1 and α3 subunit of Na+,K+-ATPase were rabbit polyclonal antibody against human ATP1A1 (55187, Proteintech, 1:1000) and ATP1A3 (sc-374050, Santa Cruz Biotechnology, 1:1000), respectively. Other primary antibodies including rabbit polyclonal antibody against human Src (#2109S, 1:1000), phosphor-Src(Tyr416) (#6943S, 1: 1000); PI3K p85 (#4257P, 1:1000), phosphor-PI3K p85(Tyr458)/p55(Tyr199) (#4228P, 1: 600), Akt (#9272, 1:1000), phosphor-Akt(Ser473) (#4060S, 1:800), Bax (#2772S, 1:1000), Pro-caspase-9 (#9502S, 1:800), Caspase-3 (#9662S, 1:800); PARP (#9542, 1:1000) and GAPDH (#2118, 1:1000) were all purchased from Cell Signaling Technology. After TBS washes, blots were then incubated with HRP-linked goat anti-rabbit IgG (#7074, 1:1000) secondary antibody for 2 h at room temperature at a 1:1000 dilution and then visualized using chemiluminescence (Pierce Biotechnology, Rockford, IL, USA).

### Assay of caspase-3 activity

The activity of caspase-3 was determined using a caspase-3 activity assay kit (C1115, Beyotime Biotechnology, Shanghai, China) according to the manufacturer's instructions. Briefly, after treatment with BF or BF211 at different concentrations for 24 h, the A549 cells were collected, washed with PBS and incubated with lysis buffer on ice for 15 min. After centrifugation at 2,000 g for 15 min at 4°C, the cell lysates were collected, and their protein concentrations were determined using the A280 method with a UV-Vis Spectrophotometer Q5000 (Quawell Technology, San Jose, CA, USA). Cell lysates (50 μg protein) were then added to reaction buffer containing the caspase-3 substrate Ac-DEVD-p-nitroanilide (2 mM) in 96-well microtiter plates and incubated at 37°C for 4 h. The caspase-3 activity in the samples was quantified by detecting the release of p-nitroanilide from Ac-DEVD-p-nitroanilide with a microplate spectrophotometer at 405 nm. The caspase-3 activity was expressed as the fold of enzyme activity in comparison with the control.

### Statistical analysis

Student’s t-test was used to evaluate the differences between the treated and the control groups. The data are expressed as the mean ± SEM, and the results from the three independent experiments were used for the statistical analysis. The asterisks indicate a significant difference (p < 0.05) compared with the untreated control.

## Results

### Effects of BF and BF211 on cell proliferation

As shown in Fig 1B, after treating A549 cells with increasing concentrations of BF or BF211 for 24 or 48 h, the viability of cells decreased in a concentration-dependent manner. The IC_50_ value of BF was 22.00 ± 3.53 nM for the 24 h treatment and 10.20 ± 1.01 nM for the 48 h treatment. The IC_50_ value of BF211 was 6.76 ± 0.41 nM for the 24 h treatment and 4.01 ± 2.68 nM for the 48 h treatment. Compared with BF, BF211 exhibited stronger inhibitory effects on cell proliferation. The proliferation-inhibiting effects of BF and BF211 in other human lung cancer cell lines, including NCI-H460, NCI-H522 and NCI-H1299 cells, are shown in S1 Fig and S2 Table (IC50 values). The results also suggested that BF211 exhibited stronger anti-proliferative effects than BF in these cells.

### Effects of BF and BF211 in inducing cell apoptosis

Representative results of the AnnexinV-FITC/PI double-labeled flow cytometry analysis of apoptosis is shown in Fig 1C. Both BF and BF211 could induce apoptosis in A549 cells after 24 h treatment in a concentration-dependent manner. The statistical results of the percentage of apoptotic cells in different groups are shown in Fig 1D. The results indicated that BF211 more strongly induced apoptosis than BF, and the difference between BF and BF211 was significant at concentrations of 25 and 50 nM.

### Acute toxicity of BF211 in mice

The graph of the survival rate of the animals receiving i.p. administration of BF211 is shown in S2 Fig. The results of acute toxicity assay of BF211 (ip.) is summarized in Table 1. The LD50 value was 14.75 mg/kg for male mice and 18.21 mg/kg for female mice. According to a previous report, the LD50 value of BF (i.p.) in mice was 2.2 mg/kg [14]. For the welfare of the animals involved, we did not repeat the whole experiment to check LD50 values of BF but only confirmed the toxicity of BF at two concentrations, 2.8 and 3.5 mg/kg. The ratios of mice that died after the administration of 2.8 mg/kg BF were 5 out of 10 male mice and 6 out of 10 female mice. The ratios of mice died after the administration of 3.5 mg/kg BF were 10 out of 10 male mice and 10 out of 10 female mice. Our results in determining the acute toxicity of BF were consistent with the reported LD50 value of BF at approximately 2.2 mg/kg. Over all, these results indicated that compared with BF, BF211 exhibited a much lower toxicity in mice.

**Table 1:** Acute toxicity of BF211 in mice.

Toxicity (mice)	LD5 (95% confidence)	LD50 (95% confidence)	LD95 (95% confidence)
Male	9.52 mg/kg (7.81~11.61 mg/kg)	14.75mg/kg (13.36~16.28 mg/kg)	22.85 mg/kg (18.89~27.64 mg/kg)
Female	12.03 mg/kg (10.04~14.41 mg/kg)	18.21 mg/kg (16.53~20.06 mg/kg)	27.55 mg/kg (22.56~33.66 mg/kg)

### Anti-cancer effects of BF and BF211 in nude mice

**Fig 2:**
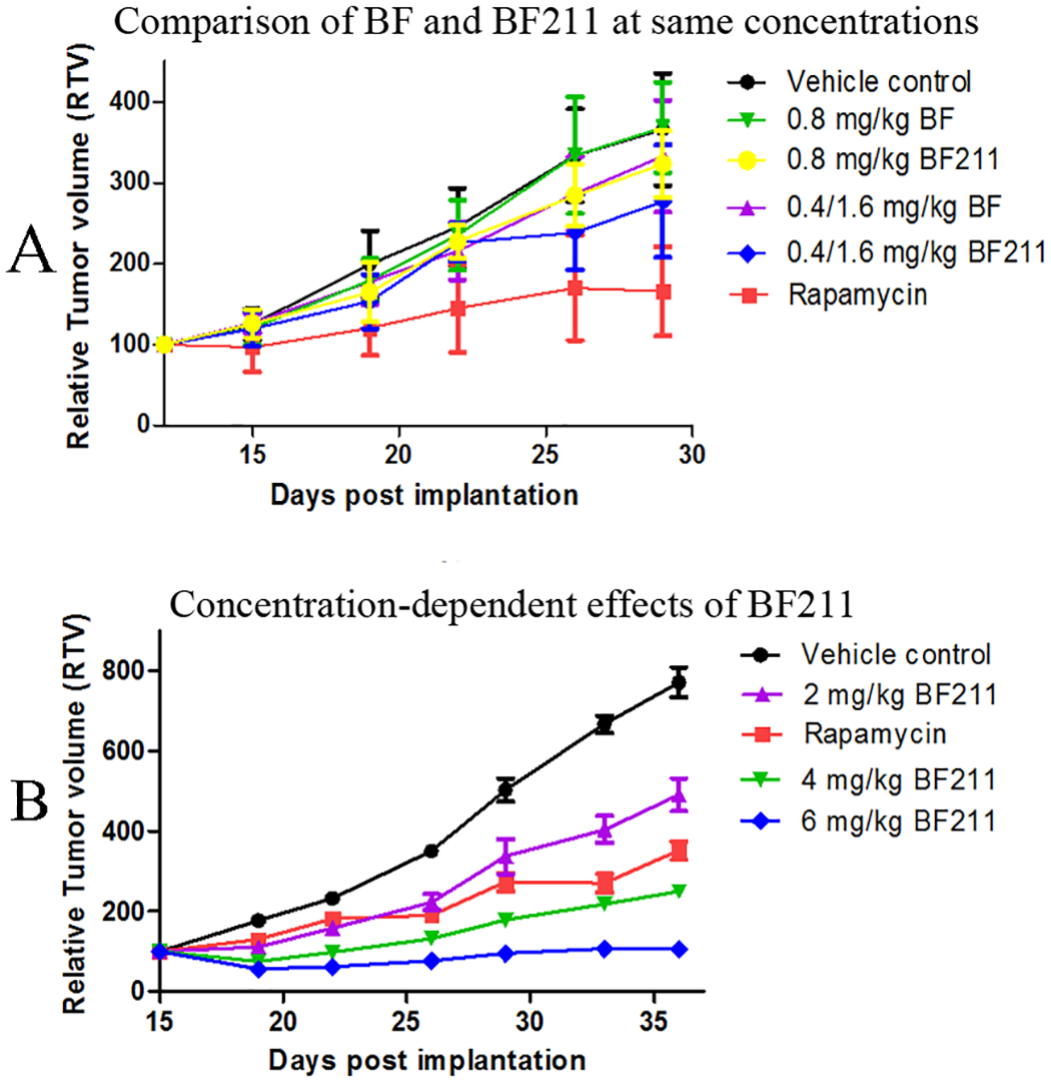
*In vivo* anti-cancer effects of BF and BF211 in nude mice inoculated with A549 cells. (A) The tumor growth curve of nude mice treated with vehicle control, rapamycin (positive control), BF or BF211 at similar concentrations as indicated. (B) The tumor growth curve of nude mice treated with vehicle control, rapamycin (positive control), and BF211 at concentrations of 2, 4, and 6 mg/kg.

The *in vivo* anti-cancer effects of BF and BF211 were checked using nude mice inoculated with A549 cells. In the first experiment comparing the effects of BF and BF211 at the same concentrations, concentrations less than 2 mg/kg for both compounds were used because of the high toxicity of BF (Fig 2A). As shown in Fig 2A, both BF and BF211 exhibited weak inhibitive effects on tumor growth and the effects of BF211 were a little stronger than that of BF at the same concentrations. In the second experiment probing the concentration-dependent effects of BF211, concentrations higher than 2 mg/kg could be used because of the low toxicity of BF211. As shown in Fig 2B, BF211 at 2, 4, and 6 mg/kg exhibited concentration-dependent inhibitive effects on the growth of tumor xenografts. The tumor growth was almost totally inhibited by BF211 at a concentration of 6 mg/kg.

### Expression of Na+/K+-ATPase α subunits in A549 cells

**Fig 3:**
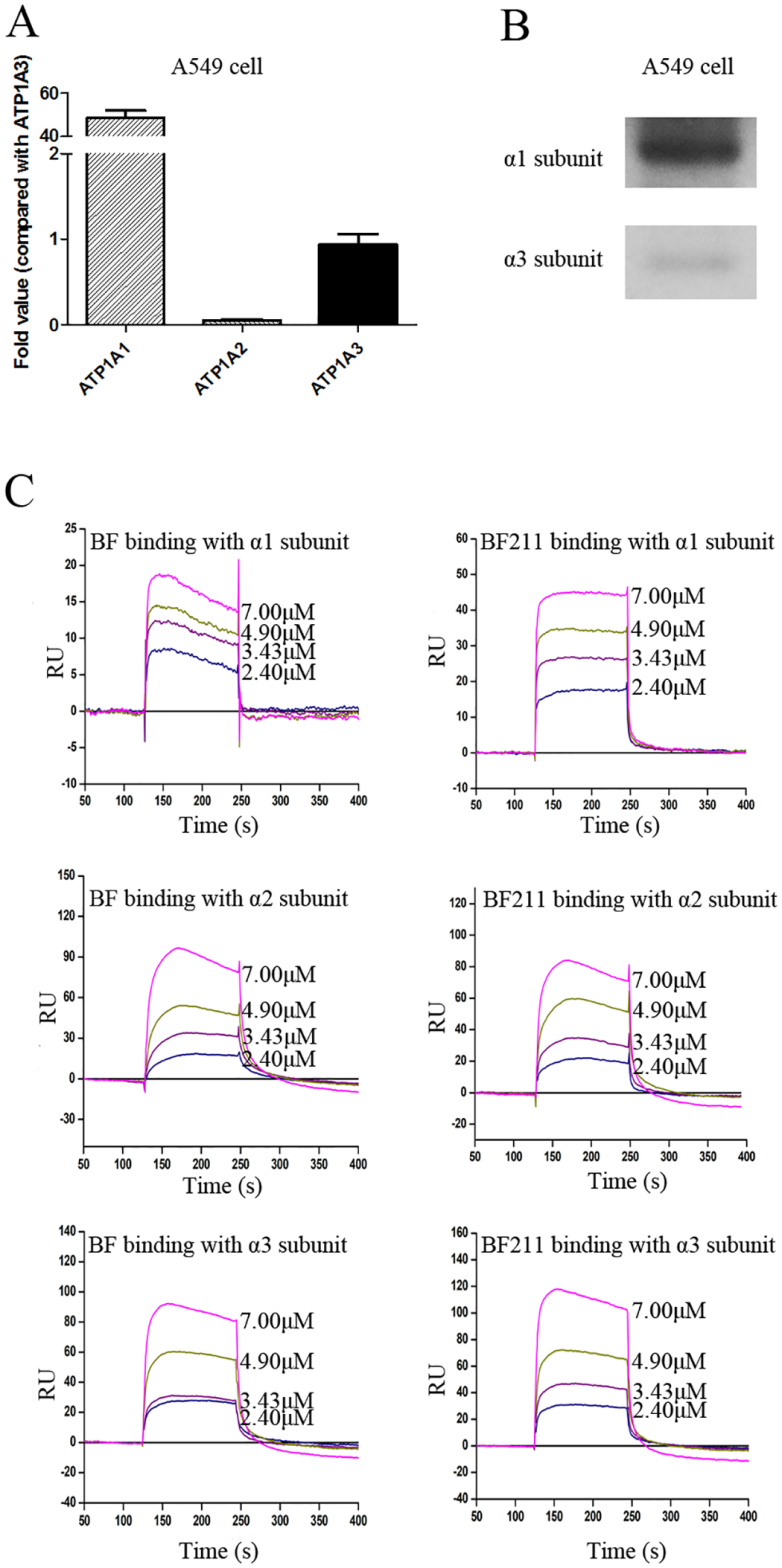
Expression of Na+/K+-ATPase α subunits in A549 cells and binding affinity of BF or BF211 to Na+/K+-ATPase α subunits. (A) The results of the RT-PCR analysis of the expression levels of the α subunits of Na+/K+-ATPase in A549 cells. The data comprise the statistical results (n = 3, mean ± SEM) of three independent experiments. (B) The representative results of the western blot assay probing the protein levels of the α1 and α3 subunits of Na+/K+-ATPase in A549 cells. (C) The real time binding affinity measurements of BF or BF211 to the recombinant Na+/K+-ATPase α subunits. The binding ability between the compound and the recombinant protein is reflected in recorded response unit (RU) values.

As shown in Fig 3A, the expression levels of ATP1A1 (encoding the α1 subunit), ATP1A2 (encoding the α2 subunit) and ATP1A3 (encoding the α3 subunit) in A549 cells were compared using the ATP1A3 gene as a normalization control (ATP1A3 gene expression level was set to equal 1). The results showed that the expression of the α2 subunit was very low, while the expression of the α1 subunit was rather high in A549 cells. The results of western blot assay of the protein levels of the α1 subunit and the α3 subunit in A549 cells are shown in Fig 3B. The protein level of α2 subunit was too low to be clearly detected by the western blot assay (data not shown). The levels of the Na+/K+-ATPase α subunits in A549 cells decrease as follows: α1>α3>α2 subunit.

### Binding affinity of BF or BF211 to Na+/K+-ATPase α subunits

The binding ability of BF or BF211 toward the recombinant proteins of α1, α2, and α3 subunits of Na+/K+-ATPase was reflected by the RU values recorded directly by the Biacore 3000 instrument. As shown in Fig 3C, the RU increased with increasing BF and BF211 concentrations, which indicated that both BF and BF211 could bind to the recombinant proteins in a concentration-dependent manner. The association (*k*_a_), dissociation (*k*_d)_, and equilibrium dissociation (*K*_D_) constants of binding between BF and BF211 with the proteins are shown in Table 2. As shown in Table 2, for the α1 protein, the *K*_D_ value of BF211 was similar to that of BF. For the α2 protein, the *K*_D_ value of BF211 was higher than that of BF, which suggested that the binding affinity of BF211 for the α2 protein was lower than that of BF. On the contrary, for the α3 protein, the *K*_D_ value of BF211 was lower than that of BF, which suggested that the binding affinity of BF211 for the α3 protein was higher that of BF. Furthermore, the fact that the *k*_a_ and *k*_d_ of the binding of BF211 with the α1 or α2 proteins were much higher than that of BF suggested that BF211 might associate and dissociate more rapidly than BF. The results suggested that there might be difference between BF and BF211 in both their binding affinity and binding kinetics in their capability of binding to the α subunits of Na+/K+-ATPase. It is noteworthy that because the α1 subunit is the major α subunit of Na+/K+-ATPase in A549 cells, the difference of BF and BF211 in the binding kinetics to the α1 subunit might contribute greatly to the difference in their effects on A549 cells.

**Table 2:** Parameters of binding between BF or BF211 with α1, α2 or α3 recombinant subunit protein of Na+/K+-ATPase.

Parameters	BF			BF211		
	α1	α2	α3	α1	α2	α3
Association (*k*_a_) (M^-1^S^-1^)	4.05	3.29	2.68e^4^	1.77e^4^	9.82e^3^	3.07e^4^
Dissociation (*k*_d_) (S^-1^)	1.01e^-5^	1.01e^-5^	1.47e-^1^	5.26e^-2^	4.41e^-2^	1.16e^-1^
Equilibrium dissociation (*K*_D_) (μM)	2.5	3.05	5.49	2.96	4.49	3.78

### Effects of BF and BF211 on the activity of porcine cortex Na+/K+-ATPase

**Fig 4:**
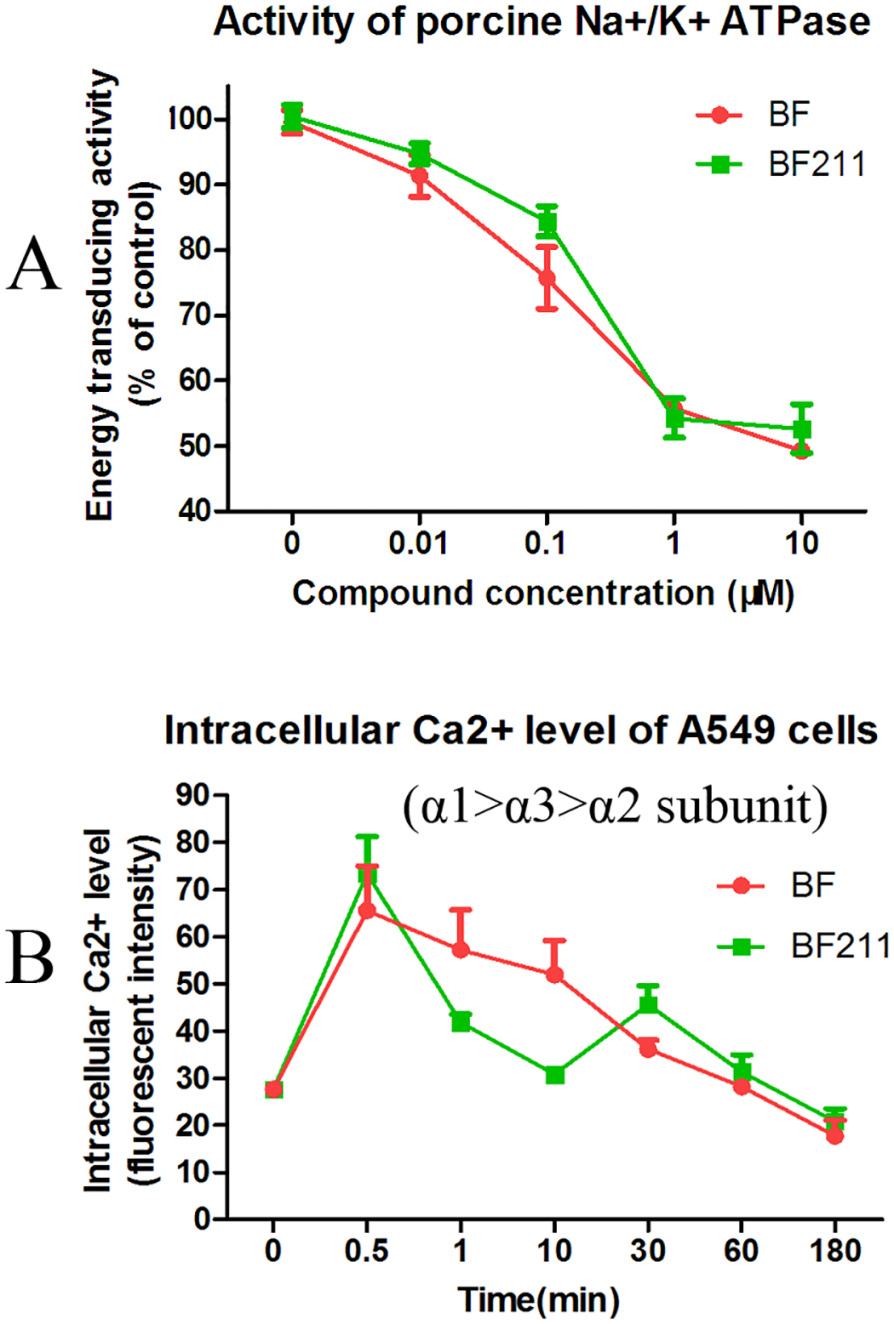
Inhibitive effects of BF or BF211 on the activity of porcine cortex Na+/K+-ATPase and the intracellular Ca2+ level of A4549 cells. (A) The activity of Na^+^/K^+^-ATPase extracted from porcine cerebral cortex in the presence of BF or BF211 at different concentrations was determined. The data are the statistical results (n = 3, mean ± SEM) of three independent experiments. (B) The level of intracellular Ca2+ level in A549 cells treated with 50 nM BF or BF211 for different time periods. The data are the statistical results (n = 3, mean ± SEM) of three independent experiments.

As shown in Fig 4A, both BF211 and BF could inhibit the activity of porcine cortex Na+/K+-ATPase in a concentration-dependent manner. Generally, the effects of BF211 and BF were similar. However, the effects of BF211 might be slightly weaker than BF at some concentrations, such as 0.1 μM.

### Time-dependent effects of BF and BF211 on the intracellular Ca2+ level of A549 cells

As shown in Fig 4B, both BF211 and BF could induce an increase in intracellular Ca2+ level of A549 cells in a concentration-dependent manner based on the inhibition of the activity of membrane Na^+^/K^+^-ATPase. In our results, the peak Ca2+ level appeared at only 0.5 min after treatment. The possibility that the actual peak level of Ca2+ might be earlier than 0.5 min could not be excluded, because we did not observe any time point earlier than 0.5 min. In cells treated with BF, the Ca2+ level decreased gradually from 0.5 to 180 min after treatment and went back to normal. In cells treated with BF211, the Ca2+ level decreased relative quickly from 0.5 to 10 min, but it then increased again and reached another peak at 30 min. Then, there was a gradual decrease to a normal level. These results suggested that BF and BF211 exhibited differences in the kinetics of inhibiting Na^+^/K^+^-ATPase in A549 cells.

### Time-dependent effects of BF and BF211 on the activation of Src in A549 cells

**Fig 5:**
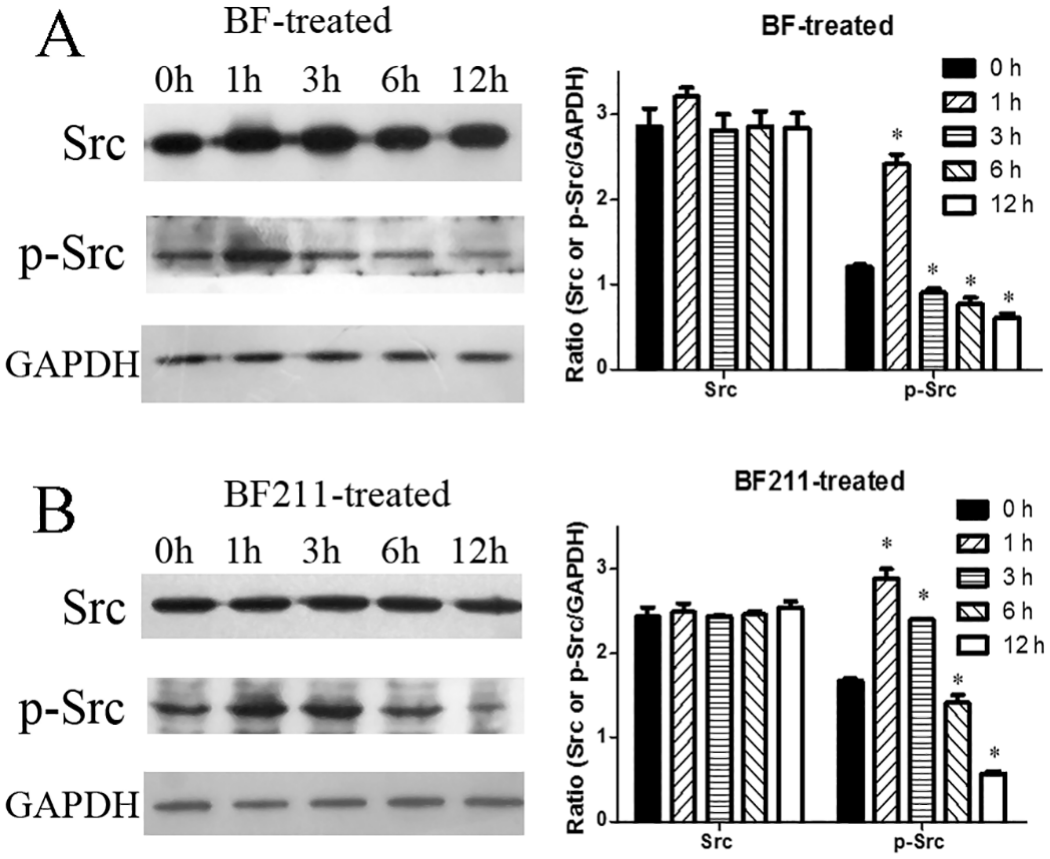
Effects of BF or BF211 on activation of Src in A549 cells. (A) The time-dependent effects of BF on the activation of Src in A549 cells treated with 50 nM BF for different time periods. Both representative western blot assay results and quantification analysis results were shown. The quantification data are the statistical results (n = 3, mean ± SEM) of three independent experiments. *p<0.05 vs. control (0 h group). (B) The time-dependent effects of BF211 on the activation of Src in A549 cells treated with 50 nM BF211 for different time periods. Both the representative western blot assay results and the quantification analysis results are shown. The quantification data comprise the statistical results (n = 3, mean ± SEM) of three independent experiments. *p<0.05 vs. control (0 h group).

The time-dependent effects of BF and BF211 on Src activation are shown in Fig 5A and 5B, respectively. Both the representative results and quantification analysis results are presented. As shown in Fig 5A, BF treatment quickly induced a significant increase in phosphorylated Src (p-Src) at 1 h after treatment, and then, the level of p-Src decreased. BF211 induced a significant increase in the level of p-Src at both 1 h and 3 h after treatment (Fig 5B). These results suggested that there might be differences in the time-dependent effects of BF and BF211 on Src activation, which indicated the activation of the Na+/K+-ATPase signalosome.

### Concentration-dependent effects of BF and BF211 on caspase-3 activation, PARP cleavage and caspase-3 enzyme activity in A549 cells

**Fig 6:**
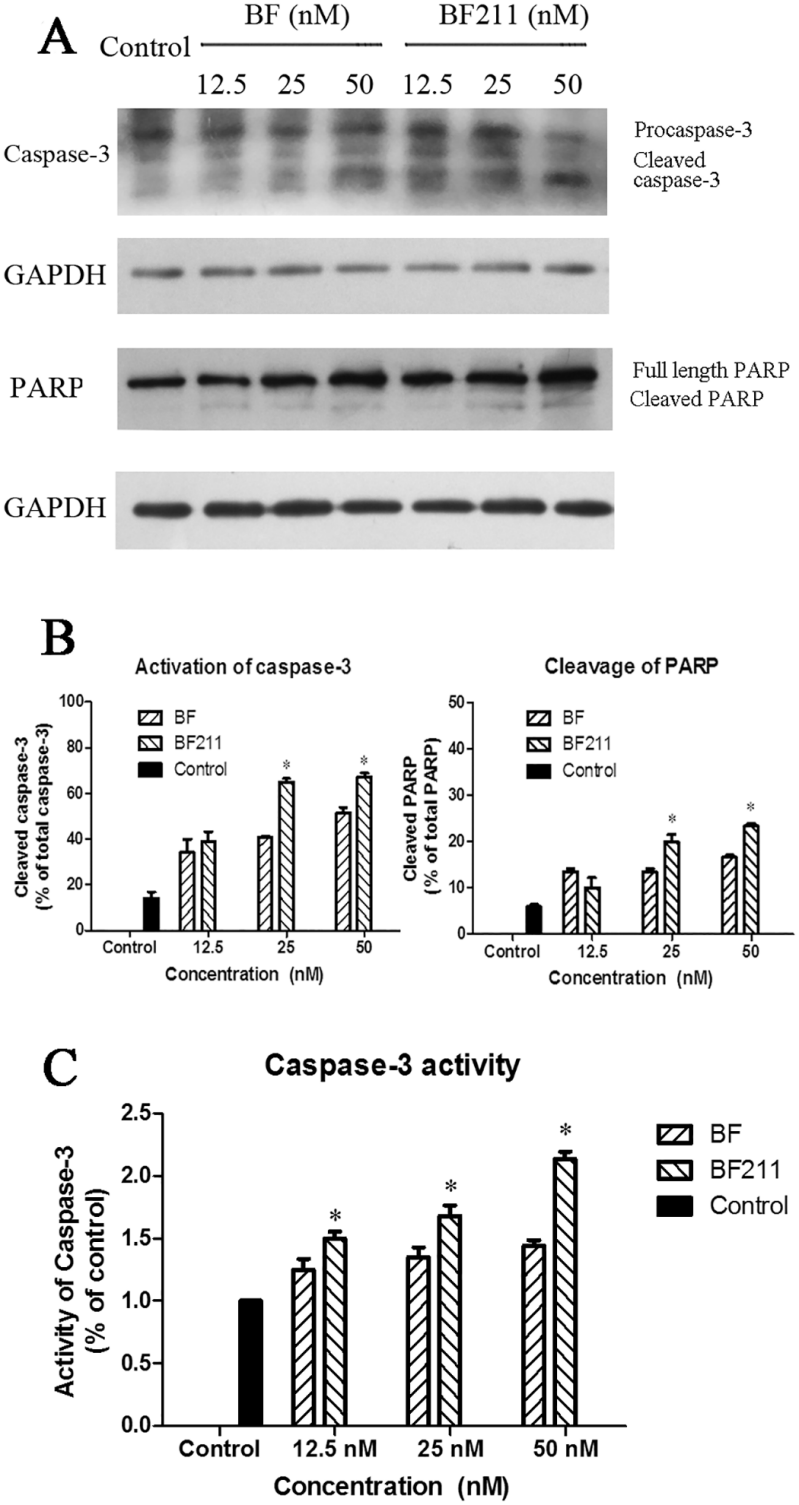
Effects of BF or BF211 on caspase-3 activation and PARP cleavage in A549 cells. (A) **The r**epresentative western blot assay results probing the levels of caspase-3 and PARP in cells treated with BF or BF211 at different concentrations for 24 h. (B) The quantification of the western blot assay results of the activation of caspase-3 (percentage of cleaved caspase-3 from the total caspase-3) and cleavage of PARP (percentage of cleaved PARP from the total PARP) in cells treated with BF or BF211 at different concentrations for 24 h. The data are the statistical results (n = 3, mean ± SEM) of three independent experiments. *p<0.05 vs. BF-treated group. (C) The activity of caspase-3 in cells treated with BF or BF211 at different concentrations for 24 h. The data comprise the statistical results (n = 3, mean ± SEM) of three independent experiments. *p<0.05 vs. BF-treated group.

The concentration-dependent effects of BF and BF211 on the activation of caspase-3 (cleavage of procaspase) and the cleavage of PARP are shown in Fig 6A (representative results) and Fig 6B (quantification analysis results). As shown in Fig 6A and 6B, both BF and BF211 could induce the activation of caspase-3 and the cleavage of PARP. The inducing effects of BF211 on apoptosis-related signal cascades were stronger than that of BF at high concentrations. The enzyme activity of caspase-3 in cells treated with BF and BF211 is shown in Fig 6C, which shows that both BF and BF211 could induce an increase in the activity of caspase-3 in a concentration-dependent manner, while the effects of BF211 were significantly stronger than that of BF.

## Discussion

Epidemiologic and experimental studies have shown the promising anti-cancer effects of cardiac steroids [6,15,16]. Several clinical trials (NCT02138292, NCT01887288 NCT02212639) related to the use of digoxin alone or in combination with other anti-cancer agents are currently recruiting participants. A clinical trial of PBI-05204 (NCT02329717), an oleander extract containing mainly oleandrin, as a new anti-cancer agent is also in progress. Furthermore, great efforts had been devoted to the development of derivatives of cardiac steroids for anti-cancer use [17,18]. Up to now, no natural cardiac steroids or their derivatives have been approved as anti-cancer agents in the United States or other Western countries. The main limitation of the anti-cancer use of cardiac steroids is the toxicity resulting from their cardiac tonic effects.

In China, the traditional Chinese medicine ChanSu which contains mainly bufadienolides, has been used as an anti-cancer agent for a long time. Huachansu injection, a water-soluble extract of ChanSu, is presently used in China in the clinic to treat patients with non-small-cell lung cancer, hepatocellular carcinoma, etc. [2]. The anti-cancer effects of BF, one of the main components of ChanSu, have been extensively reported [19,20,21,22,23,24]. To find suitable candidates for new drug development, we synthesized hundreds of BF derivatives and probed their anti-cancer effects as well as their toxicity. Fortunately, a compound called BF211 was found to be a promising compound with stronger anti-cancer effects and lower toxicity. Lung cancer is one of the most prevalent malignancies worldwide, and most cases are non-small cell lung cancer. The median overall survival of patients with advanced stage non-small lung cancer treated with the current standard chemotherapy is only approximately 10 months [25]. In the present study, our results showed that both BF and BF211 could potently inhibit the proliferation of cultured lung non-small-cell cancer A549 cells and induce apoptosis in the cells. The cytotoxic effects of BF211 are stronger than that of BF. In nude mice inoculated with A549 cells, BF211 also exhibited slightly stronger inhibitive effects on tumor growth than BF at the same concentration. Importantly, because the acute toxicity of BF211 was much lower than that of BF, BF211 could be used at higher concentrations for anti-cancer therapy, which suggested that BF211 has a much wider therapy window. At higher concentrations (4 or 6 mg/kg), BF211 strongly inhibited the growth of tumor xenografts. These results suggested the potency of developing BF derivatives, such as BF211, as new anti-cancer agents.

We conducted further studies to clarify the possible mechanistic differences between BF and BF211 in terms of their anti-cancer effects and acute toxicity. Up until now, Na^+^/K^+^-ATPase was the only well-accepted direct target of cardiac steroids. The α subunits (α1, α2, α3, α4) of Na+/K+-ATPase are the active subunits involved in the binding of cardiac steroids and Na+/K+-ATPase. The fact that the distribution of α subunits are tissue- and species-specific suggests the particular functions of each subunit. Generally, the α1 subunit is expressed ubiquitously, while the α2 subunit is present largely in muscle, heart, brain, etc. [26]. The α3 subunit was found almost exclusively in neurons and ovaries, but it also occurs in white blood cells and the hearts of some species; however, the α4 subunit is only expressed in sperm and is specifically synthesized at the spermatogonia stage [26]. Notably, the expression levels of the α1 and/or α3 subunit are high in tumor tissues [27,28]. Our results also showed that the level of the α1 subunit in A549 cells was much higher than the other α subunits.

Within the plasma membrane, there are two pools of Na^+^/K^+^-ATPase, he classical pool of the enzyme acting as an energy transducing ion pump and t the signal transducing pool of the enzyme that is restricted to the caveolae, forming the so-called “Na^+^/K^+^-ATPase signalosome” [29]. The inhibition of the energy transducing ion pump is the basis of the cardiac tonic effects of cardiac steroids. On the contrary, the mechanism by which cardiac steroids exert their anti-cancer effects is generally attributed to induced changes in the Na+/K+-ATPase signalosome, which contains Na+/K+-ATPase, Src, PI3K, etc., and activate of cell death-related cascades [15,30].

In the present study, BF211 exhibited lower toxicity than BF in mice. Several studies have shown that the cardiac tonic effects of cardiac steroids are mainly mediated by theα2 subunit of Na+/K+-ATPase [31,32,33]. While previous reports showed that both the α1 and α2 subunits could be detected in adult rat hearts, rat α2 subunit, with a higher affinity to cardiac steroids, is less abundant than the α1 subunit, which has a very low affinity for cardiac steroids [34]. Therefore, both the α1 and α2 subunits might be involved in the toxicity of BF and BF211 in mice. Interestingly, when the activity of porcine cortex Na+/K+-ATPase, which expresses the levels of the α subunits in the order α2>α3>α1 [35] is inhibited, BF211 exhibited slightly weaker effects than BF at some concentrations, such as at 0.1 μM. The weaker effects of BF211 on porcine Na+/K+-ATPase might be consistent with the weaker binding affinity of BF211 to the α2 subunit found in the Biacore assay. Furthermore, the results of the Biacore assay in the present study also showed that though the binding affinities of BF and BF211 to the α1 protein were similar, the binding kinetic parameters of BF211 were different from those of BF. With much higher values for the association parameter *k*_*a*_ and the dissociation parameter *k*_*d*_, BF211 might associate and dissociate more rapidly than BF in its binding with α1 protein. The residence time of the receptor-ligand complexes has been found to be very important in the biological functions of ligands [36]. In the present study, we also observed the time-dependent effects of BF or BF211 on the intracellular Ca2+ level of A549 cells. The increase in the intracellular Ca2+ level in cells treated with cardiac steroids resulted from the inhibition of cell membrane Na+/K+-ATPase. In A549 cells, which express the α subunits in the order α1>α3>α2, the decrease in the intracellular Ca2+ level after the peak at 0.5 min in BF211-treated cells occurred more quickly than that of BF-treated cells. The appearance of another peak in the intracellular Ca2+ level of BF211-treated cells was interesting, although its reason for occurring requires further study. Over all, the difference in the binding affinities and kinetics of BF211 and BF to the α subunits of Na+/K+-ATPase might both contribute to their differences in acute toxicity in mice.

In the present study, BF211 exhibited stronger cytotoxicity than BF in A549 cells. Previous studies have reported that BF could induce apoptosis in cancer cells [21,22,23]. The flow cytometry analysis results of the present study showed that the percentage of apoptotic cells in cells treated with BF211 was higher than that of the cells treated with BF at the same concentration. Furthermore, the western blot assay results also showed that the activation of apoptosis-related signal cascades, such as caspase-3 activation and PARP cleavage, was also stronger in cells treated with BF211 than in cells treated with BF at the same concentration. The activity of caspase-3 was also significantly higher in BF211-treated cells than in BF-treated cells. The stronger inducing effects of BF211 on signaling cascades, such as the Src pathway, and down-stream apoptosis-related cascades might be the basis of its higher cytotoxicity in A549 cells, compared with BF cytotoxicity. The results of probing time-dependent influence of BF and BF211 on the phosphorylation of Src indicated that both BF and BF211 could induce the activation of Src, but there was a difference in the kinetics of the change in level of p-Src in BF-treated or BF211-treated cells. Consistent with the high expression of the α1 and/or α3 subunit of Na+/K+-ATPase in cancer cells, previous reports have suggested that the α1 and/or α3 subunit play a critical role in the effects of cardiac steroids on cancer cells [28,37,38]. The α1 subunit was found to be the major α subunit of Na+/K+-ATPase in A549 cells in the present study. The differences in binding kinetics of BF or BF211 to the α1 subunit of Na+/K+-ATPase might contribute to their differences in activation of the Na+/K+-ATPase signalosome, such as the phosphorylation of Src.

In summary, we demonstrated that BF211 is a potent active anti-cancer compound with a lower toxicity compared with BF. It inhibited the proliferation and induced apoptosis in A549 cancer cells through the activation of signaling cascades downstream of the Na+/K+-ATPase signalosome. The stronger anti-cancer effects and lower toxicity of BF211 in mice compared with those of BF might be based on its specific binding characteristics for the Na+/K+-ATPase. The identification and characterization of a cardiac steroid derivative that exhibits stronger anti-cancer effects and lower toxicity than the natural compound might have important implications for the development of novel anti-cancer agents from cardiac steroids.

## Supporting Information

S1 Fig Inhibiting effects of BF or BF211 on proliferation of H460, H522 and H1299 cells. Cell viability (MTT assay result) of cells treated with various concentrations of BF or BF211 for 48 h. Data were statistical results of three independent experiments.
(PDF)

S2 Fig Survival rate of mice received i.p. administration of BF211 at different doses. Totally 140 mice were randomly assigned into 14 groups (10 mice in each group) to receive single doses of 9.4, 11.1, 13.1, 15.4, 18.1, 21.3 and 25 mg/kg of BF211 for male mice and single doses of 9.8, 11.5, 13.6, 16.0, 18.8, 22.1 and 26 mg/kg of BF211 for female mice.
(PDF)

S1 Table Sequence of primers used in RT-PCR analysis. (PDF)

S2 Table IC50 values of BF and BF211 (72 h treatment) in inhibiting proliferation of human lung cancer cell lines. (PDF)
