# Supplementary material for: A Novel Bufalin Derivative Exhibited Stronger Apoptosis-Inducing Effect than Bufalin in A549 Lung Cancer Cells and Lower Acute Toxicity in Mice
Source: PLoS One. 2016 Jul 26;11(7):e0159789. doi: 10.1371/journal.pone.0159789 (PMC4961401; doi:10.1371/journal.pone.0159789)
Supplement: S1 Fig — Cell viability (MTT assay result) of cells treated with various concentrations of BF or BF211 for 48 h. Data were statistical results of three independent experiments. (PDF) [file pone.0159789.s001.pdf]

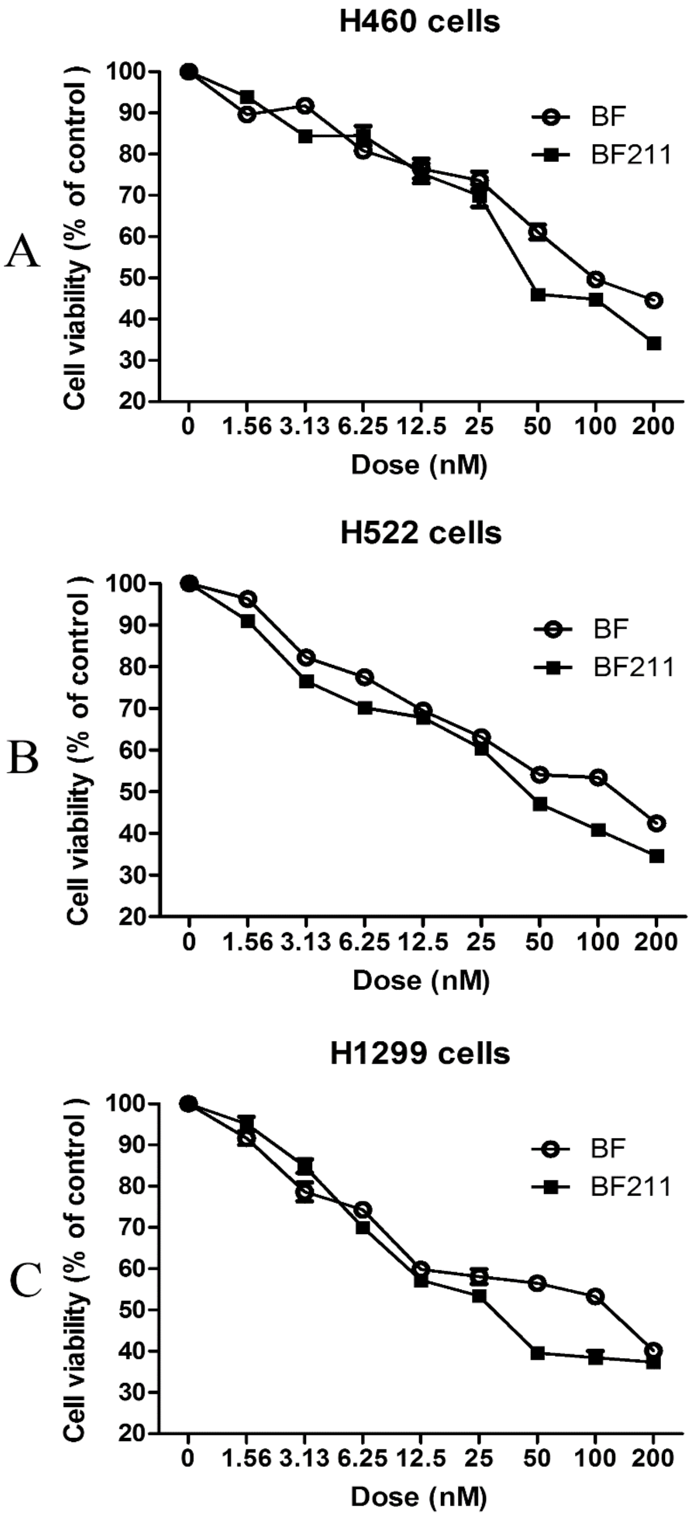

S1 Fig. Inhibiting effects of BF or BF211 on proliferation of H460, H522 and H1299 cells. Cell viability (MTT assay result) of cells treated with various concentrations of BF or BF211 for 48 h. Data were statistical results of three independent experiments.
