# Supplementary material for: A Novel Bufalin Derivative Exhibited Stronger Apoptosis-Inducing Effect than Bufalin in A549 Lung Cancer Cells and Lower Acute Toxicity in Mice
Source: PLoS One. 2016 Jul 26;11(7):e0159789. doi: 10.1371/journal.pone.0159789 (PMC4961401; doi:10.1371/journal.pone.0159789)
Supplement: S2 Fig — Totally 140 mice were randomly assigned into 14 groups (10 mice in each group) to receive single doses of 9.4, 11.1, 13.1, 15.4, 18.1, 21.3 and 25 mg/kg of BF211 for male mice and single doses of 9.8, 11.5, 13.6, 16.0, 18.8, 22.1 and 26 mg/kg of BF211 for female mice. (PDF) [file pone.0159789.s002.pdf]

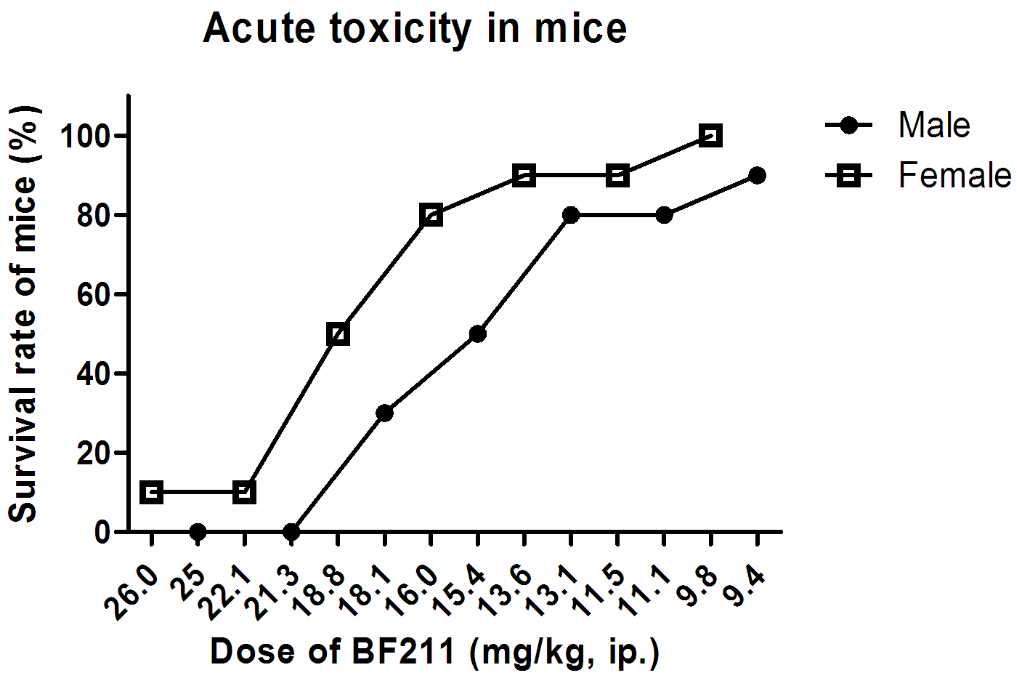

S2 Fig. Survival rate of mice received i.p. administration of BF211 at different doses. Totally 140 mice were randomly assigned into 14 groups (10 mice in each group) to receive single doses of 9.4, 11.1, 13.1, 15.4, 18.1, 21.3 and 25 mg/kg of BF211 for male mice and single doses of 9.8, 11.5, 13.6, 16.0, 18.8, 22.1 and 26 mg/kg of BF211 for female mice.
