# Supplementary material for: A Novel Bufalin Derivative Exhibited Stronger Apoptosis-Inducing Effect than Bufalin in A549 Lung Cancer Cells and Lower Acute Toxicity in Mice
Source: PLoS One. 2016 Jul 26;11(7):e0159789. doi: 10.1371/journal.pone.0159789 (PMC4961401; doi:10.1371/journal.pone.0159789)
Supplement: S1 Table — (PDF) [file pone.0159789.s003.pdf]

**S1 Table.** Sequence of primers used in RT-PCR analysis

| Gene | Sequence (5'-3') |
|---|---|
| ATP1A1 | Forward: CTGTGGATTGGAGCGATTCTT |
| | Reverse: TTACAACGGCTGATAGCACCA |
| ATP1A2 | Forward: CACCACCGAAGATCAGTCTGG |
| | Reverse: CGCTTAGACACGGAGATGTTC |
| ATP1A3 | Forward: AAGGAGGTGGCTATGACAGAG |
| | Reverse: GTGAGTGCGTTAGGCCCAT |
