# Supplementary material for: A Novel Bufalin Derivative Exhibited Stronger Apoptosis-Inducing Effect than Bufalin in A549 Lung Cancer Cells and Lower Acute Toxicity in Mice
Source: PLoS One. 2016 Jul 26;11(7):e0159789. doi: 10.1371/journal.pone.0159789 (PMC4961401; doi:10.1371/journal.pone.0159789)
Supplement: S2 Table — (PDF) [file pone.0159789.s004.pdf]

S2 Table. IC50 values of BF and BF211 (72 h treatment) in inhibiting proliferation of human lung cancer cell lines

Cell lines	IC50 values (nM)	
	BF	BF211
NCI-H460	114.29 ± 11.19	68.00 ± 6.91
NCI-H522	98.69 ± 2.76	48.29 ± 0.69
NCI-H1299	88.31 ± 11.87	40.08 ± 4.04
